# Effect of Data Reduction Techniques on Daily Moderate to Vigorous Physical Activity Collected with ActiGraph^®^ in People with COPD

**DOI:** 10.3390/jcm12165340

**Published:** 2023-08-16

**Authors:** Patrícia Rebelo, Joana Antão, Dina Brooks, Alda Marques

**Affiliations:** 1 Lab3R—Respiratory Research and Rehabilitation Laboratory, School of Health Sciences (ESSUA), University of Aveiro, 3810-193 Aveiro, Portugal; patriciarebelo@ua.pt (P.R.); joanaantao@ua.pt (J.A.); 2 iBiMED—Institute of Biomedicine, University of Aveiro, 3810-193 Aveiro, Portugal; 3 Department of Medical Sciences, University of Aveiro, 3810-193 Aveiro, Portugal; 4 Department of Research and Development, Ciro, 6085 NM Horn, The Netherlands; 5 Department of Respiratory Medicine, Maastricht University Medical Centre, NUTRIM School of Nutrition and Translational Research in Metabolism, Faculty of Health, Medicine and Life Sciences, Maastricht University, 6200 MD Maastricht, The Netherlands; 6 School of Rehabilitation Science, Faculty of Health Sciences, McMaster University, Hamilton, ON L8S 1C7, Canada; brookd8@mcmaster.ca; 7 West Park Healthcare Centre, Toronto, ON M6M 2J5, Canada

**Keywords:** accelerometer, processing criteria, physical activity, chronic respiratory diseases

## Abstract

ActiGraph^®^ is a valid, frequently used, accelerometer to quantify moderate to vigorous physical activities (MVPA) in people with COPD. The impact of ActiGraph processing techniques on this population is unknown. This study aimed to explore the effect of data reduction techniques on MVPA in people with COPD. MVPA/day, through ActiGraph GT3X+, was estimated using: Troiano, Freedson 98 and FreedsonVM3 cutoffs, 15-s and 60-s epochs, and normal and low-frequency extension (LFE) filters. Cutoff, epoch, and filter effects were explored with Aligned Rank Transform-ANOVA. Lin’s concordance correlation coefficients and Bland–Altman plots were used to evaluate agreement and bias between different techniques. The analysis included 136 people with COPD (79% male; 68 ± 8 years; FEV1 51 ± 17% predicted). MVPA/day differed according to cutoff, filter, and epoch selection (*p*-value < 0.001). FreedsonVM3 cutoff, 15-s epochs, and LFE yielded the highest MVPA (45 min/day, 68% of physically active participants). Troiano cutoff, 60-s epochs, and normal filter yielded the lowest MVPA (8 min/day, 20% of physically active participants). Only comparisons between Troiano and Freedson98 cutoffs presented an almost perfect agreement. ActiGraph data reduction techniques affected MVPA/day estimates and their interpretation at the individual and group level. Studies using different processing criteria should not be compared in people with COPD. Future studies with a gold standard are required to ascertain which processing technique produces the most accurate MVPA estimates in COPD. Meanwhile, future trials employing the ActiGraph GT3X+ may consider estimating MVPA based on Freedson VM3 cutofffs, 60-s epochs, and normal filter.

## 1. Introduction

Physical activity (PA) is intimately related to the general health status of people with chronic obstructive pulmonary disease (COPD) and to the disease prognosis [1,2]. Monitoring time spent in moderate to vigorous physical activities (MVPA) is, therefore, important to ensure people with COPD comply with the World Health Organization (WHO) PA recommendations and maximize PA-related benefits [3]. Among the different objective instruments available to measure MVPA [1], we can highlight ActiGraph GT3X+^®^ (ActiGraph, Pensacola, FL, USA), one of the most frequently used accelerometers in COPD [4]. ActiGraph GT3X+ is a triaxial accelerometer that presents several advantages: It is a small, non-invasive device, easily used by participants in free-living conditions, relatively affordable, and has been recognized as a valid and feasible tool to assess PA in people with COPD [5,6,7].

Time spent in MVPA is derived from one of the following ActiGraph outputs: Vertical axis counts, resulting from the sum and conversion of acceleration data [8], or vector magnitude (VM), pooled using counts from the three axes [9]. Time spent in MVPA represents the sum of each minute in which counts/VM surpassed a previously established cutoff [10]. A wide variety of cutoffs has been proposed to estimate MVPA in adults and older healthy populations [11,12], however, none has been validated for people with COPD. An overview of studies published in COPD [2,13,14,15,16,17,18], showed three cutoffs being used to define MVPA in this population: (i) Troiano cutoff, which uses vertical axis counts and was established according to different studies using a treadmill or track walking protocols [19], (ii) Freedson98 cutoff, that also uses vertical axis counts and was developed from a treadmill protocol [20], and (iii) FreedsonVM3 cutoffs, developed by the same group as Freedson98 cutoff applying a similar methodology, but using VM [21]. Criteria to choose among the different cutoffs in COPD are unknown.

During the processing of accelerometer data to estimate MVPA, the assessor must also choose the amount of time to sum the raw data, i.e., the epoch length [22]. ActiGraph GT3X+ data can be processed using various epoch lengths, from 1 to 240 s. There is no consensus on which epoch length should be used to estimate MVPA in COPD. In fact, recommendations for using 15-s epoch lengths to measure free-living PA in COPD [4] and for choosing the same epoch length as the one reported in the cutoff validation study [11] are found in the literature.

Another data reduction decision to define MVPA is choosing between the normal and low-frequency extension (LFE) filters [23]. On ActiLife, the ActiGraph software, the normal filter is activated by default. Nevertheless, the use of the LFE filter has been recommended for slower movers’ populations since it was specifically designed to increase ActiGraph sensitivity to detect low-intensity PA [12,23]. PA in people with COPD is usually characterized by reduced intensity movements than matched healthy individuals [24]. Thus, LFE could be more suitable than the normal filter. Nevertheless, it should be noted that the three cutoffs that have been used in COPD were developed using the normal filter. Applying the LFE filter will alter the VM/counts, and to what extent this affects MVPA estimates in people with COPD is still unclear.

Studies investigating PA in people with COPD have employed different processing criteria [13,16,17]. These discrepancies may lead to different MVPA results, prevent comparisons among studies, and may mislead PA recommendations in COPD. Thus, the aim of this study was to examine the effect of different ActiGraph data reduction techniques, i.e., cutoff, epoch length, and filter selection, on daily MVPA in people with COPD.

## 2. Materials and Methods

This was an observational retrospective, cross-sectional and secondary study integrated into three large trials (NCT03799666; NCT04223362; NCT04711057). This study included people with COPD (FEV1/FVC < 0.7), clinically stable in the previous month, who were referred to community-based pulmonary rehabilitation programs conducted in the Respiratory Research and Rehabilitation Laboratory—Lab3R, School of Health Sciences, University of Aveiro and in seven primary health-care centers from the Center region of Portugal, between January 2019 and May 2022. Only participants who used the ActiGraph GT3X+ to assess PA were included. This study was approved by the Ethics Committees from Unidade Investigação em Ciências da Saúde—Enfermagem (Ref. P620-10/2019), Centro Hospitalar Baixo Vouga (Ref. 15-05-2019), Administração Regional de Saúde do Centro (Ref. 73/2016 and 16/2020), and the National Committee for Data Protection (no. 7295/2016). Informed consent was obtained from all participants, and privacy was assured according to the European Union General Data Protection Regulation 2016/679 (GPDR). This report follows the strengthening reporting of observational studies in epidemiology (STROBE) guidelines for cross-sectional studies.

### 2.1. Outcome and Data Reduction Techniques

The outcome measure explored in this study was daily time spent in MVPA measured with ActiGraph GT3X+ (ActiGraph, Pensacola, FL, USA). Participants were instructed to wear the ActiGraph on the right side of the waist (above the iliac crest) for 7 consecutive days and to remove it only during activities that involved water, such as showering or swimming. ActiGraph data was collected using a 30 Hz sample frequency and analyzed using ActiLife v6.10.4 (ActiGraph, Pensacola, FL, USA).

Participants’ PA data were considered valid and included in the analysis if participants had at least 4 days with a minimum of 8 h (480 min) of wearing time [7]. Choi algorithm was used to define non-wear time (i.e., a minimum of 90 min with zero counts, with an allowance for an interruption of two minutes with non-zero counts, ensuring that on the 30-min window before and after that interruption, there must be zero counts) [25]. The analysis included ActiGraph data collected before pulmonary rehabilitation as default. Post-pulmonary rehabilitation accelerometer data were included only for participants who met the wearing time criteria at that timepoint but not at the pre-pulmonary rehabilitation assessment. Participants were categorized as physically active or inactive, according to the WHO recommendations (≥150 min of weekly time on MVPA) [3]. This study explored the influence of the data reduction criteria outlined in Table 1.

### 2.2. Data Analysis

Data analysis and graphs were performed/created using R 4.2.0 (The R Foundation, Indianapolis, IN, USA). QQ-plot inspection was used to explore data distribution. The effect of cutoff, filter, and epoch selection was analyzed using Aligned Rank Transform (ART) ANOVA, appropriate for non-parametric variables. The level of significance was set at 0.05. Two sensitivity analyses, including exclusively participants with ActiGraph data from the pre-pulmonary rehabilitation or the post-pulmonary rehabilitation assessment were conducted. To perform post-hoc pairwise comparisons on daily MVPA, obtained through different cutoffs, filters, and epochs, we fixed two factors (i.e., filter and epoch, cutoff and epoch, cutoff and filter), thus, resulting in 24 pairs of measurements. Bonferroni correction was used to calculate *p*-values for pairwise comparisons (α = 0.05/24 = 0.002). Concordance among the different data reduction techniques was explored with Lin’s concordance correlation coefficient, which was interpreted as almost perfect when above 0.99, substantial between 0.95 and 0.99, moderate between 0.9 and 0.95, and poor if lower than 0.9 [26]. Bland–Altman plots were created, and bias (mean difference) and 95% limits of agreement were calculated. Bias was considered significant if zero was not within the 95% confidence interval of the mean difference (represented as a grey shadow). Proportional bias was assessed using linear regression and considered relevant whenever the slope was significant (*p*-value < 0.05).

## 3. Results

**Figure 1:**
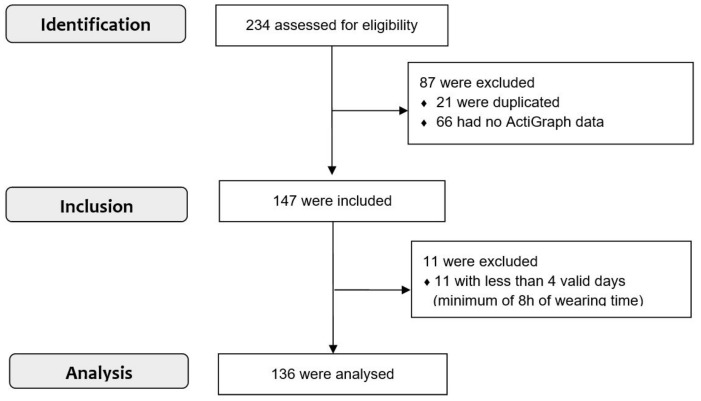
Flow diagram of participants with chronic obstructive pulmonary disease recruited and included in the study.

ActiGraph data of 147 people with COPD was screened. PA data from 11 participants did not fulfil the wear time validation criterion, thus, 136 people with COPD (121 [89%] with pre-pulmonary rehabilitation data and 15 [11%] with post-pulmonary rehabilitation data) were included in the analysis (Figure 1).

Participants were, on average, 69 ± 8 years old, presented a mean FEV1 of 51 ± 17% predicted, they were mostly male (79%), and pertained to GOLD grades 2 (42%) and 3 (44%), and group B (57%). All participants’ characteristics are presented in Table 2.

Daily wear time for the 136 participants ranged from 1055 [796; 1298] when using normal filter, to 1167 [799; 1370] min/day, when applying the LFE filter, with, on average, 7 valid days. Daily MVPA results according to different data reduction techniques are presented in Table 3.

Processing criteria affected daily MVPA in people with COPD and a 3-way interaction effect was present (*p*-value < 0.001 for cutoff, filter and epoch effects, and for the interaction between filter:epoch, filter:cutoff, epoch:cutoff, and filter:epoch:cutoff) (Table S1 from the Supplementary Materials). Results were similar across the two sensitivity analyses performed (Tables S2 and S3 from the Supplementary Materials). Within the 24 pairwise comparisons explored, 20 were statistically different from each other (*p*-value < 0.001), and four were not (Table 3). When comparing cutoffs, Troiano and Freedson98 resulted in similar daily MVPA estimates, whilst both significantly differed from FreedsonVM3. The lowest daily MVPA estimates were yielded by Troiano cutoff (ranging from 8 to 18 min/day, with 20 to 38% of participants being considered physically active) and the highest by FreedsonVM3 cutoff (ranging from 15 to 45 min/day, with 36 to 68% participants being considered physically active). Additionally, normal filter (ranging from 8 to 32 min/day, with 20 to 61% participants being considered physically active) vs. LFE (ranging from 9 to 45 min/day, with 24 to 68% participants being considered physically active), and 60-s (ranging from 8 to 23 min/day, with 20 to 44% participants being considered physically active) vs. 15-ss epochs (ranging from 15 to 45 min/day, with 33 to 68% participants being considered physically active) resulted in statistically different daily MVPA estimates, with LFE and 15-s epochs consistently yielding higher estimates.

Concordances, bias, and upper and lower limits of agreement for the 24 pairwise comparisons are presented in Table 4.

**Figure 2:**
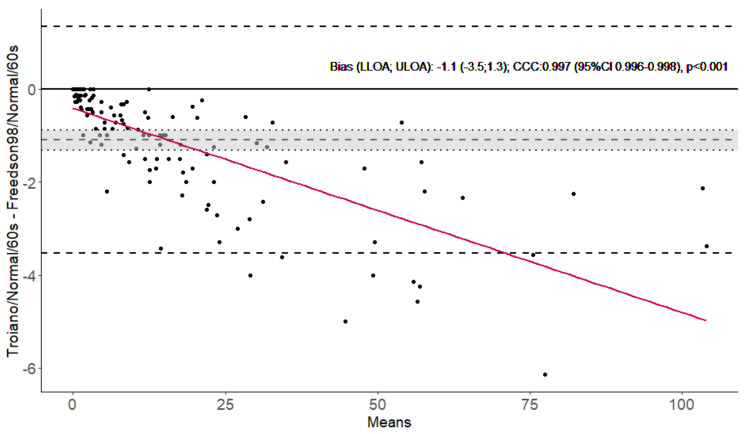
Bland–Altman 95% limits of agreement plot for Troiano and Freedson 98 cutoffs, with 60-s epoch and normal filter fixed in people with chronic obstructive pulmonary disease (*n* = 136). Dashed lines represent bias (mean difference), lower and upper limits of agreement, and the grey shadow represents the 95% confidence interval of the mean difference. Bias, lower and upper limits of agreement, Lin’s correlation coefficient and the *p*-value for the slope of the linear regression of the differences in averages (red line) are presented. CCC—Lin’s concordance correlation coefficient. CI—Confidence interval; LLOA—lower limit of agreement; ULOA—upper limit of agreement.

**Figure 3:**
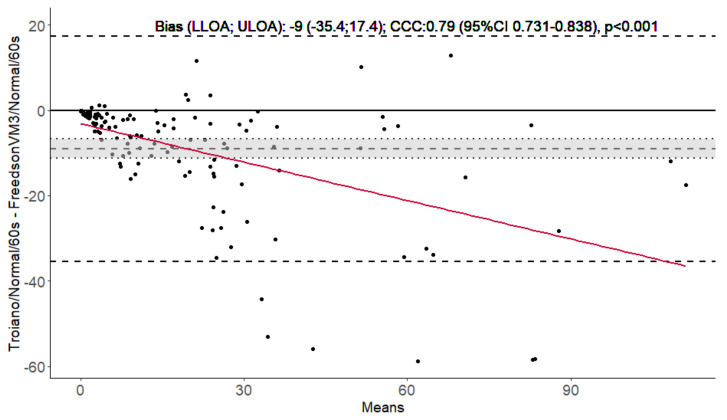
Bland–Altman 95% limits of agreement plot for Troiano and Freedson VM3 cutoffs, with 60-s epoch and normal filter fixed in people with chronic obstructive pulmonary disease (*n* = 136). Dashed lines represent bias (mean difference), lower and upper limits of agreement, and the grey shadow represent the 95% confidence interval of the mean difference. Bias, lower and upper limits of agreement, Lin’s correlation coefficient, and the *p*-value for the slope of the linear regression of the differences in averages (red line) are presented. CCC—Lin’s concordance correlation coefficient. CI—Confidence interval; LLOA—lower limit of agreement; ULOA—upper limit of agreement.

**Figure 4:**
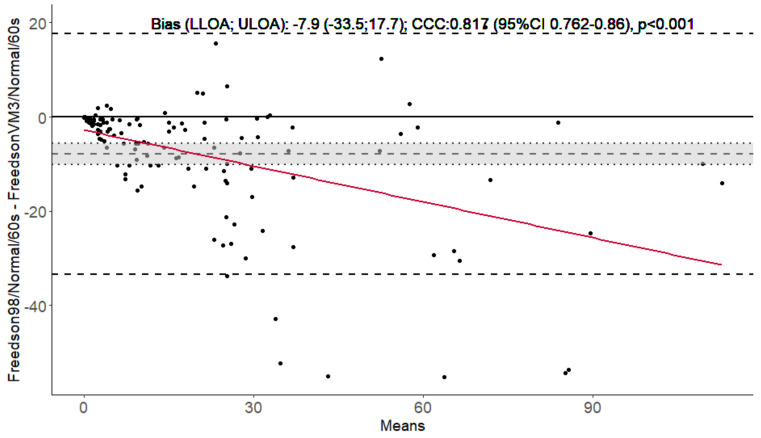
Bland–Altman 95% limits of agreement plot for Freedson 98 and Freedson VM3 cutoffs, with 60-s epoch and normal filter fixed in people with chronic obstructive pulmonary disease (*n* = 136). Dashed lines represent bias (mean difference), lower and upper limits of agreement, and the grey shadow represent the 95% confidence interval of the mean difference. Bias, lower and upper limits of agreement, Lin’s correlation coefficient, and the *p*-value for the slope of the linear regression of the differences in averages (red line) are presented. CCC—Lin’s concordance correlation coefficient. CI—Confidence interval; LLOA—lower limit of agreement; ULOA—upper limit of agreement.

Three illustrative Bland–Altman 95% limits of agreement plots comparing the 3 cutoffs (60-s epoch and normal filter were fixed) are represented in Figure 2, Figure 3 and Figure 4, and the remaining 21 are presented in the Supplementary Materials.

Ten of the pairwise comparisons presented poor concordances, six were moderate, four substantial, and four almost perfect. Bias was considered significant for all 24 comparisons, and it increased as time in MVPA increased (proportional bias with a *p*-value < 0.001 for all linear regression slopes) (Figure 2, Figure 3, Figure 4 and Figures S1–S21 in the Supplementary Materials). Concordances were almost perfect, and bias was low (less than 2 min/day), when Troiano and Freedson98 cutoffs were compared (using the same epoch and filter). Conversely, all comparisons between FreedsonVM3 and Troiano, or Freedson98, cutoffs were poor and presented high bias (more than 8 min/day). Comparisons between epoch lengths consistently presented higher bias (e.g., FreedsonVM3-60 s-Normal vs. FreedsonVM3-15 s-Normal had a bias of 16 min/day) than comparisons between filters (e.g., FreedsonVM3-60 s-Normal vs. FreedsonVM3-60 s-LFE had a bias of 7 min/day). Additionally, comparisons using LFE, or 15-s epochs, also presented higher bias and lower concordances than the ones using normal or 60-s epochs (e.g., FreedsonVM3-60 s-Normal vs. Troiano-60 s-Normal had a bias of 9 min/day, against the bias of 26 min/day yielded by the comparison of FreedsonVM3-15 s-LFE vs. Troiano-15 s-LFE).

## 4. Discussion

This study found that cutoff, epoch length, and filter selection influence daily MVPA estimates measured with ActiGraph GT3X+ during free-living conditions in people with COPD. Daily MVPA varied greatly across the different data reduction techniques, from 8 to 45 min/day, which, in turn, resulted in a highly discordant prevalence of physically active participants, ranging from 20 to 68%. Therefore, MVPA estimates produced by different ActiGraph data reduction techniques in people with COPD are not comparable. Additionally, the selection of data reduction technique will affect MVPA interpretation at the individual and group level, thus potentially misleading tailored interventions and policymakers when targeting PA needs.

Troiano and Freedson 98 presented similar daily MVPA estimates, almost perfect concordances, and bias was below two min/day, but both were different from estimates yielded by Freedson VM3 cutoff, which generally doubled the results (bias increased up to 26 min/day). In fact, Freedson VM3 categorized 15% to 29% more participants as physically active. Differences found among cutoffs may be explained by the ActiGraph models used during the cutoff validation studies. Whilst Troiano and Freedson 98 cutoffs were developed using a uniaxial ActiGraph (model 7164), which considered only counts from the vertical axis [19,20], Freedson VM3 cutoffs were established using ActiGraph GT3X and used VM, i.e., data from the three axes [21]. The use of VM instead of only vertical axis data has been recommended to analyze PA and sedentary behavior in COPD [5,27]. Similar results when comparing MVPA cutoffs have been reported, with Freedson VM3 always eliciting higher MVPA estimates than Freedson 98 (10 and 16 min/day more in MVPA when compared to Freedson 98) [18,28] and comparable MVPA estimates being found when Troiano and Freedson 98 cutoffs are used, regardless of the epoch length (differences below 2 min/day) [29], in studies including healthy adults. It should be noted that in these studies, refs. [18,28,29] the amount of time spent in MVPA was consistently greater compared to the registered in our sample. In fact, using Freedson VM3, processed using 60-s epochs and normal filter, the MVPA of healthy individuals ranged from 21 min in females [28] to 39 min in males [18], whereas in our study, people with COPD presented approximately 15 min of MVPA.

In this study, concordances between the two epoch lengths were poor to moderate, and bias ranged from 7 to 20 min/day. The 15-s epochs produced larger daily MVPA estimates and classified 13 to 25% more participants as physically active than the 60-s. These findings are consistent with other studies on healthy adults and children, which showed that usually, time in MVPA increases as epoch length decreases [29,30,31,32]. Nevertheless, there is evidence that the epoch length effect varies according to the nature of PA. Specifically, variations are expected in: (i) The intensity of PA, with longer epoch lengths resulting in larger MVPA estimates if PA intensity is high and lower MVPA estimates when intensity is low (e.g., running vs. walking) [33], (ii) the duration and type of PA assessment, with longer epochs resulting in larger MVPA estimates during single and structured PAs, but in smaller estimates during longer-period daily living assessments [34], and (iii) the type of MVPA analysis, with longer epoch lengths yielding larger MVPA estimates in bout-accumulated analysis (e.g., MVPA on 10-min bouts), but smaller estimates when simply all minutes spent in MVPA are summed [29]. Short epoch lengths have been recommended to accurately assess PA behavior, in free-living conditions, characterized by intermittent and brief movements such as in COPD [11,12,32,35]. However, when using the three cutoffs explored in this study, researchers should be aware that their validation studies used 60-s epochs, and so far, no study has established their validity using different epoch lengths.

Regarding the filter effect, concordances between filters were substantial to almost perfect, and bias ranged from 2 to 11 min/day. The LFE filter yielded higher daily MVPA estimates than the normal filter and classified 3 (using Freedson98 and 60-s epochs) to 8% (using FreedsonVM3 and 60-s epochs) more participants as physically active. These results are aligned with previous research including healthy adults and children [36,37,38,39]. When using the LFE filter, a lower bound for human movement detection is applied, compared to the normal filter [23], thus resulting in higher vertical counts or VM and, consequently, in more time spent in MVPA. The LFE outperformed the normal filter when analyzing sedentary behavior in people with COPD [27]. Considering the low-intensity PA nature of people with COPD [24], we could argue that the LFE would be preferred to the normal filter.

Proportional bias (higher amounts of MVPA lead to greater bias than lower MVPA) was consistently present across all the compared processing techniques. A similar pattern of accelerometer measurement error related to PA level has been previously identified [12]. The larger discrepancy between techniques when MVPA increases can be related to the growing error observed in ActiGraph readings with higher PA intensities [40,41].

Previous studies in people with COPD have shown that triaxial accelerometers are more valid than uniaxial accelerometers, [5,6] thus, Freedson VM3 (which estimates MVPA using data from 3 axes) [21] is recommended in comparison with the Troiano or Freedson 98 cutoffs (which use data from the vertical axis) [19,20]. We could then hypothesize that Freedson VM3, using 15-s epochs (appropriate to intermittent PA patterns) [11,32] and LFE filter (advised for slower movers) [23], would produce the best MVPA estimates in people with COPD. Nevertheless, this would imply changing the cutoff and filter used in the Freedson VM3 original study (60-s and normal filter) [21], which, as demonstrated in this study, would affect MVPA estimates, thus compromising the cutoff validity. In addition, the normal filter has been shown to be more accurate in deriving steps per day in older adults than the LFE filter [11,42]. Ensuring that the same ActiGraph processing technique is suitable for both MVPA and steps per day, two widely used PA outcome measures in COPD, would streamline research and enhance its applicability in clinical practice. Given the lack of studies validating ActiGraph cutoffs or proposing specific cutoffs for people with COPD, as well as the absence of a consensus on which ActiGraph data reduction methods should be used, [12] we, therefore, suggest future trials employing the ActiGraph GT3X+ to estimate MVPA based on Freedson VM3 cutofffs, 60-s epochs, and normal filter. The MVPA estimate yielded using this data reduction technique (nearly 15 min/day) was similar to that presented in other COPD studies using the same methodology [17].

Finally, in line with the recommendations provided for objectively measured PA in COPD, ref. [7] our results emphasize the importance of future trials accurately and comprehensively reporting the data reduction techniques used. The absence of this information may hinder the comparability of MVPA across studies and mislead PA recommendations in people with COPD.

### Study Limitations and Strengths

This study presents some limitations and strengths that should be recognized. First, the absence of a criterion measure prevents us from knowing which data reduction technique is more accurate and establishing the clinical relevance of the bias found. Secondly, we only included three cutoffs and two epoch lengths, even though several other cutoffs have been proposed for healthy adults [11], and ActiLife encompasses numerous epoch lengths. Additionally, we did not analyze the influence of the non-wear algorithm selection (e.g., Troiano algorithm [19] vs. Choi algorithm [25]). Even realizing that our analysis does not cover the full range of possible ActiGraph processing decisions, we chose to limit the analysis to the approaches that have been used in COPD to ensure readability and facilitate comprehension of our results. Thirdly, there is a potential risk for selection bias, as the sample was recruited from community pulmonary rehabiliation programs, mainly composed of males and participants pertaining to the GOLD stages 2 and 3 and group B. This hampers the generalizability of our results, as PA levels in people with COPD seem to be influenced by disease severity, namely GOLD grades, [43] and the proportional bias verified in our study supports that higher MVPA estimates will lead to higher bias than lower MVPA. Finally, compliance with the PA guidelines was assessed using an absolute intensity approach, as the three cutoffs explored were developed in healthy adult individuals [19,20,21]. Thus, time spent in MVPA was estimated without considering numerous individual factors that differentiate people with COPD from healthy adults, such as age, altered body composition, and higher energy requirement to perform their daily activities, [24,44,45,46,47,48], all of which impact PA intensity [47,49,50,51]. A more suitable approach for this population would likely involve relative intensity to estimate MVPA. Future studies developing specific accelerometer cutoffs tailored to people with COPD or exploring alternative methodologies that combine relative intensity and accelerometry are urgently required.

Nevertheless, it is important to acknowledge that this is the first study exploring the impact of different data reduction techniques with ActiGraph, one of the most frequently used accelerometers, to estimate daily MVPA in people with COPD, an outcome measure that is crucial to establish compliance with PA guidelines. This study included a large sample of people with COPD, thus, strengthening our confidence in the findings reported.

## 5. Conclusions

The current study showed that data reduction techniques, i.e., cutoffs, epoch lengths, and filter selection, impact daily MVPA estimates, measured with ActiGraph GT3X+, during free-living conditions in people with COPD. Specifically, MVPA estimates are higher when Freedson VM3, 15-s epochs, and LFE filter are chosen, compared with Freedson or Troiano cutoffs, 60-s epochs, and normal filter. Future trials employing the ActiGraph GT3X+ may consider estimating MVPA based on Freedson VM3 cutofffs, 60-s epochs, and normal filter. Nevertheless, studies ascertaining which ActiGraph data reduction technique produces more accurate MVPA estimates in COPD are needed to then enable standardization and comparability among studies. Until then, clinicians, researchers, and policymakers should be aware that current MVPA estimates using ActiGraph may be hampering accurate interpretation of PA levels and potentially misleading proper interventions or policies targeting people with COPD.

## Figures and Tables

**Table 1:** ActiGraph data reduction techniques explored.

Data Reduction Technique		Description	Reference
Cutoff to calculate MVPA	Troiano	MVPA ≥ 2020 vertical axis counts	Troiano et al. [19]
	Freedson 98	MVPA ≥ 1952 vertical axis counts	Freedson et al. [20]
	Freedson VM3	MVPA ≥ 2690 VM	Sasaki et al. [21]
Epoch length	60-s	Following the recommendation to choose the same epoch length as the one reported in the cutoff validation studies	Migueles et al. [11]
	15-s	Following the recommendation to use 15-s epochs to measure free-living PA in COPD	Byrom et al. [4]
Filter	Normal	Activated by default	ActiGraph Corporation [23]
	LFE	Designed to detect lower amplitude movements over the normal filter	ActiGraph Corporation [23]

Legend: COPD: Chronic Obstructive Pulmonary Disease; LFE: Low frequency extension filter; MVPA: Moderate to vigorous physical activities; PA: Physical activity; VM: Vector magnitude.

**Table 2:** Sample characterization (*n* = 136).

Characteristics	People with COPD
Age, years	68.7 ± 8.3
Sex, male, *n* (%)	107 (78.7)
BMI, kg/m^2^	27.1 ± 5.3
Smoking status, current/former/never, *n* (%)	23 (17)/82 (60.7)/30 (22.2)
Packs/year	31.5 [10; 70]
Exacerbations/year ^a^	0 [0; 1]
FEV_1_, L/%predicted	1.3 ± 0.5/50.7 ± 17
FVC, L/%predicted	2.5 ± 0.9/74.2 ± 18.8
FEV_1_/FVC, %	52.8 ± 11.4
GOLD grades 1/2/3/4, *n* (%)	8 (6)/56 (41.8)/59 (44)/11 (8.2)
GOLD groups, A/B/E, *n* (%)	37 (27.2)/78 (57.4)/21 (15.5)
Charlson Comorbidity Index, mild/moderate/severe, *n* (%)	16 (11.9)/80 (59.3)/39 (28.9)
Medication, *n* (%)	
SABA	17 (12.5)
SAMA	8 (5.9)
LABA	16 (11.8)
LAMA	41 (30.1)
LAMA/LABA combination	43 (31.6)
ICS	17 (12.5)
ICS/LABA combination	46 (33.8)
ICS/LABA/LAMA combination	14 (10.3)
LTRA	11 (8.1)
Xanthines	10 (7.4)
Expectorants	17 (12.5)
mMRC, points	2 [1; 3]
CAT, points	14.7 ± 8.3
6MWD, meters	403.4 ± 99.1

Values are presented as mean ± standard deviation or median [interquartile range], unless otherwise stated. ^a^ past year. Legend: BMI: Body mass index; CAT: COPD assessment test; COPD: Chronic obstructive pulmonary disease; FEV_1_: Forced expiratory volume in one second; FVC: Forced vital capacity; GOLD: Global Initiative for chronic obstructive lung disease; ICS: Inhaled corticosteroid; LABA: Long-acting beta-agonists; LAMA: Long-acting muscarinic antagonist; LRTA: Leukotriene receptor antagonist; mMRC: Modified medical research council questionnaire; SABA: Short-acting beta-agonists; SAMA: Short-acting muscarinic antagonist; 6MWD: 6-min walk distance.

**Table 3:** Daily moderate-to-vigorous physical activity (min/day) and number and percentage of people with chronic obstructive pulmonary disease categorized as physically active, according to cutoff, filter, and epoch length selection (*n* = 136).

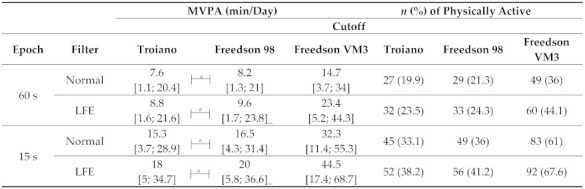

Data is presented as median [interquartile range], and number (percentage) of participants categorised as physically active (≥150 min of weekly time in moderate to vigorous physical activities). Legend: LFE: Low-frequency extension; MVPA: Moderate to vigorous physical activity; 60 s: 60-s epochs; 15 s: 15-s epochs. ^a^ Pairwise comparisons that were NOT statistically different (*p*-value > 0.002).

**Table 4:** Lin’s concordance correlation coefficient and bias, upper and lower 95% limits of agreement (min/day) for moderate to vigorous physical activities in people with chronic obstructive pulmonary disease. All analyses were performed with 2 factors fixed (epoch and filter; cutoff and filter; cutoff and epoch) (*n* = 136).

	Pair of Data Reduction Techniques		Lin’s Concordance Correlation Coefficient (95% CI)	Bias and Upper and Lower Limits of Agreement in min/Day
Comparisons between cutoffs	Troiano, 60 s, Normal	Freedson 98, 60 s, Normal	0.997 (0.996–0.998)	−1.1 (−3.5; 1.3)
		Freedson VM3, 60 s, Normal	0.79 (0.731–0.838)	−9 (−35.4; 17.4)
	Troiano, 15 s, Normal	Freedson 98, 15 s, Normal	0.997 (0.996–0.998)	−1.5 (−4; 1)
		Freedson VM3, 15 s, Normal	0.68 (0.606–0.742)	−18.1 (−54.5; 18.3)
	Troiano, 60 s, LFE	Freedson 98, 60 s, LFE	0.996 (0.995–0.997)	−1.3 (−4.1; 1.5)
		Freedson VM3, 60 s, LFE	0.705 (0.634–0.765)	−14.1 (−49.3; 21)
	Troiano, 15 s, LFE	Freedson 98, 15 s, LFE	0.997 (0.995–0.997)	−1.7 (−4.5; 1.1)
		Freedson VM3, 15 s, LFE	0.603 (0.525–0.671)	−25.5 (−69.6; 18.7)
	Freedson 98, 60 s, Normal	Freedson VM3, 60 s, Normal	0.817 (0.762–0.86)	−7.9 (−33.5; 17.7)
	Freedson 98, 15 s, Normal	Freedson VM3, 15 s, Normal	0.713 (0.642–0.772)	−16.6 (−52; 18.8)
	Freedson 98, 60 s, LFE	Freedson VM3, 60 s, LFE	0.741 (0.674–0.796)	−12.8 (−46.5; 20.9)
	Freedson 98, 15 s, LFE	Freedson VM3, 15 s, LFE	0.64 (0.563–0.705)	−23.7 (−66.5; 19.1)
Comparison between epochs	Troiano, 60 s, Normal	Troiano, 15 s, Normal	0.918 (0.984–0.937)	−7 (−19.1; 5.1)
	Troiano, 60 s, LFE	Troiano, 15 s, LFE	0.916 (0.891–0.935)	−8.2 (−20.5; 4.2)
	Freedson 98, 60 s, Normal	Freedson 98, 15 s, Normal	0.919 (0.896–0.938)	−7.4 (−19.5; 4.8)
	Freedson 98, 60 s, LFE	Freedson 98, 15 s, LFE	0.916 (0.892–0.935)	−8.6 (−21.2; 4)
	Freedson VM3, 60 s, Normal	Freedson VM3, 15 s, Normal	0.83 (0.788–0.865)	−16.1 (−36.9; 4.7)
	Freedson VM3, 60 s, LFE	Freedson VM3, 15 s, LFE	0.835 (0.794–0.868)	−19.5 (−40.8; 1.8)
Comparison between filters	Troiano, 60 s, Normal	Troiano, 60 s, LFE	0.983 (0.978–0.987)	−2 (−8.7; 4.6)
	Troiano, 15 s, Normal	Troiano, 15 s, LFE	0.986 (0.982–0.989)	−3.2 (−8.5;2.1)
	Freedson 98, 60 s, Normal	Freedson 98, 60 s, LFE	0.984 (0.978–0.988)	−2.2 (−8.9; 4.4)
	Freedson 98, 15 s, Normal	Freedson 98, 15 s, LFE	0.986 (0.981–0.989)	−3.5 (−9;2.1)
	Freedson VM3, 60 s, Normal	Freedson VM3, 60 s, LFE	0.947 (0.933–0.959)	−7.2 (−20.6; 6.3)
	Freedson VM3, 15 s, Normal	Freedson VM3, 15 s, LFE	0.946 (0.932–0.958)	−10.6 (−23.4; 2.2)

Bias was calculated as data reduction technique on the right minus technique on the left (e.g., Freedson 98, 60 s, normal—Troiano, 60 s, normal). Legend: LFE—low-frequency extension; concordance interpretation—almost perfect (>0.99) 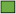; substantial (0.95–0.99) 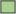; moderate (0.90–0.95) 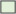; poor (<0.9) 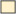.

## Data Availability

The data presented in this study are available on request from the corresponding author.

## Supplementary Materials

The following supporting information can be downloaded at: https://www.mdpi.com/article/10.3390/jcm12165340/s1, Table S1: Summary output from the Aligned Rank Transform-ANOVA with cutoff, epoch, and filter effects on estimates of time spent in moderate-to-vigorous physical activities in people with chronic obstructive pulmonary disease (*n* = 136). Table S2: Summary output from the Aligned Rank Transform-ANOVA with cutoff, epoch, and filter effects on estimates of time spent in moderate-to-vigorous physical activities including ActiGraph data from people with chronic obstructive pulmonary disease assessed before pulmonary rehabilitation (*n* = 121). Table S3: Summary output from the Aligned Rank Transform-ANOVA with cutoff, epoch, and filter effects on estimates of time spent in moderate-to-vigorous physical activities including ActiGraph data from people with chronic obstructive pulmonary disease assessed after pulmonary rehabilitation (*n* = 15). Figure S1: Bland–Altman 95% limits of agreement plot for Troiano and Freedson98 cutoffs, with 60-s epoch and low-frequency extension filter fixed in people with chronic obstructive pulmonary disease (*n* = 136). Figure S2: Bland–Altman 95% limits of agreement plot for Troiano and FreedsonVM3 cutoffs, with 60-s epoch and low-frequency extension filter fixed in people with chronic obstructive pulmonary disease (*n* = 136). Figure S3: Bland–Altman 95% limits of agreement plot for Freedson98 and FreedsonVM3 cutoffs, with 60-s epoch and low-frequency extension filter fixed in people with chronic obstructive pulmonary disease (*n* = 136). Figure S4: Bland–Altman 95% limits of agreement plot for Troiano and Freedson98 cutoffs, with 15-s epoch and normal filter fixed in people with chronic obstructive pulmonary disease (*n* = 136). Figure S5: Bland–Altman 95% limits of agreement plot for Troiano and FreedsonVM3 cutoffs, with 15-s epoch and normal filter fixed in people with chronic obstructive pulmonary disease (*n* = 136). Figure S6: Bland–Altman 95% limits of agreement plot for Freedson98 and FreedsonVM3 cutoffs, with 15-s epoch and normal filter fixed in people with chronic obstructive pulmonary disease (*n* = 136). Figure S7: Bland–Altman 95% limits of agreement plot for Troiano and Freedson98 cutoffs, with 15-s epoch and low-frequency extension filter fixed in people with chronic obstructive pulmonary disease (*n* = 136). Figure S8: Bland–Altman 95% limits of agreement plot for Troiano and FreedsonVM3 cutoffs, with 15-s epoch and low-frequency extension filter fixed in people with chronic obstructive pulmonary disease (*n* = 136). Figure S9: Bland–Altman 95% limits of agreement plot for Freedson98 and FreedsonVM3 cutoffs, with 15-s epoch and low-frequency extension filter fixed in people with chronic obstructive pulmonary disease (*n* = 136). Figure S10: Bland–Altman 95% limits of agreement plot for 60-s and 15-s epochs, with Troiano cutoff and normal filter fixed in people with chronic obstructive pulmonary disease (*n* = 136). Figure S11: Bland–Altman 95% limits of agreement plot for 60-s and 15-s epochs, with Freedson98 cutoff and normal filter fixed in people with chronic obstructive pulmonary disease (*n* = 136). Figure S12: Bland–Altman 95% limits of agreement plot for 60-s and 15-s epochs, with FreedsonVM3 cutoff and normal filter fixed in people with chronic obstructive pulmonary disease (*n* = 136). Figure S13: Bland–Altman 95% limits of agreement plot for 60-s and 15-s epochs, with Troiano cutoff and low frequency extension filter fixed in people with chronic obstructive pulmonary disease (*n* = 136). Figure S14: Bland–Altman 95% limits of agreement plot for 60-s and 15-s epochs, with Freedson98 cutoff and low frequency extension filter fixed in people with chronic obstructive pulmonary disease (*n* = 136). Figure S15: Bland–Altman 95% limits of agreement plot for 60-s and 15-s epochs, with FreedsonVM3 cutoff and low frequency extension filter fixed in people with chronic obstructive pulmonary disease (*n* = 136). Figure S16: Bland–Altman 95% limits of agreement plot for normal and low-frequency extension filters, with Troiano cutoff and 60-s epochs fixed in people with chronic obstructive pulmonary disease (*n* = 136). Figure S17: Bland–Altman 95% limits of agreement plot for normal and low-frequency extension filters, with Freedson98 cutoff and 60-s epochs fixed in people with chronic obstructive pulmonary disease (*n* = 136). Figure S18: Bland–Altman 95% limits of agreement plot for normal and low-frequency extension filters, with FreedsonVM3 cutoff and 60-s epochs fixed in people with chronic obstructive pulmonary disease (*n* = 136). Figure S19: Bland–Altman 95% limits of agreement plot for normal and low-frequency extension filters, with Troiano cutoff and 15-s epochs fixed in people with chronic obstructive pulmonary disease (*n* = 136). Figure S20: Bland–Altman 95% limits of agreement plot for normal and low-frequency extension filters, with Freedson98 cutoff and 15-s epochs fixed in people with chronic obstructive pulmonary disease (*n* = 136). Figure S21: Bland–Altman 95% limits of agreement plot for normal and low-frequency extension filters, with FreedsonVM3 cutoff and 15-s epochs fixed in people with chronic obstructive pulmonary disease (*n* = 136).
