# Supplementary material for: Effect of Data Reduction Techniques on Daily Moderate to Vigorous Physical Activity Collected with ActiGraph® in People with COPD
Source: J Clin Med. 2023 Aug 16;12(16):5340. doi: 10.3390/jcm12165340 (PMC10455487; doi:10.3390/jcm12165340)
Supplement: Supplementary file 1 [file jcm-12-05340-s001.zip › jcm-2432173-supplementary.pdf]

Supplementary material

Effect of data reduction techniques on daily moderate to vigorous physical activity collected with ActiGraph® in people with COPD

Table S1 – Summary output from the Aligned Rank Transform-ANOVA with cutoff, epoch, and filter effects on estimates of time spent in moderate-to-vigorous physical activities in people with chronic obstructive pulmonary disease (*n* = 136).

Fixed Effects	F-value	Partial eta squared	*p*-value
Filter	520.90	0.79	<0.001
Epoch length	518.79	0.79	<0.001
Cutoff	206.78	0.61	<0.001
Filter x Epoch length	135.78	0.5	<0.001
Filter x Cutoff	361	0.73	<0.001
Epoch length x Cutoff	234.57	0.63	<0.001
Filter x Epoch length x cutoff	108.42	0.45	<0.001

Table S2 – Summary output from the Aligned Rank Transform-ANOVA with cutoff, epoch, and filter effects on estimates of time spent in moderate-to-vigorous physical activities including ActiGraph data from people with chronic obstructive pulmonary disease assessed before pulmonary rehabilitation (*n* = 121).

Fixed Effects	F-value	Partial eta squared	*p*-value
Filter	563.56	0.82	<0.001
Epoch length	477.44	0.8	<0.001
Cutoff	175.73	0.59	<0.001
Filter x Epoch length	184.03	0.61	<0.001
Filter x Cutoff	309.47	0.72	<0.001
Epoch length x Cutoff	219.75	0.65	<0.001
Filter x Epoch length x cutoff	97.27	0.45	<0.001

Table S3 – Summary output from the Aligned Rank Transform-ANOVA with cutoff, epoch, and filter effects on estimates of time spent in moderate-to-vigorous physical activities including ActiGraph data from people with chronic obstructive pulmonary disease assessed after pulmonary rehabilitation (*n* = 15)

Fixed Effects	F-value	Partial eta squared	P-value
Filter	18.42	0.57	<0.001
Epoch length	38.14	0.73	<0.001
Cutoff	28.72	0.67	<0.001
Filter x Epoch length	6.57	0.32	0.023
Filter x Cutoff	26.64	0.66	<0.001
Epoch length x Cutoff	15.86	0.53	0.001
Filter x Epoch length x cutoff	8.92	0.39	<0.001

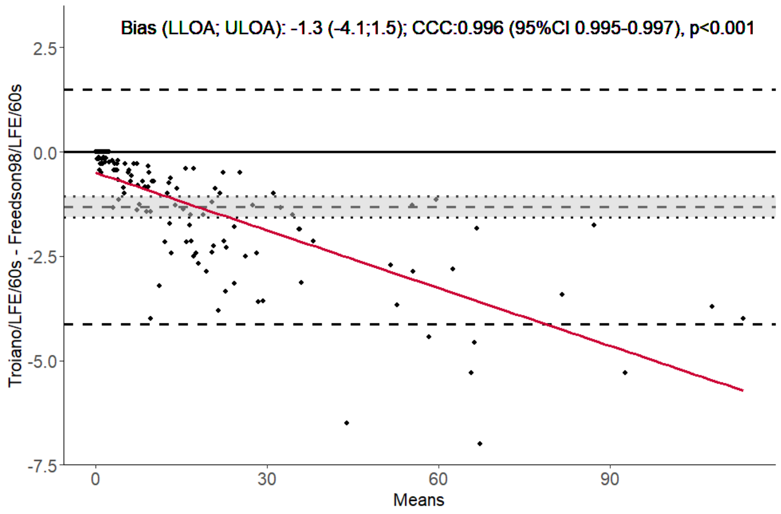

Figure S1: Bland–Altman 95% limits of agreement plot for Troiano and Freedson98 cutoffs, with 60-second epoch and low-frequency extension filter fixed in people with chronic obstructive pulmonary disease (*n*=136). Dashed lines represent bias (mean difference), lower and upper limits of agreement and the grey shadow represent the 95% confidence interval of the mean difference. Bias, lower and upper limits of agreement, Lin's correlation coefficient and the *p*-value for the slope of the linear regression of the differences on averages (red line) are presented. CCC - Lin's concordance correlation coefficient. CI – Confidence interval; LLOA – lower limit of agreement; LFE – low frequency extension filter; ULOA – upper limit of agreement.

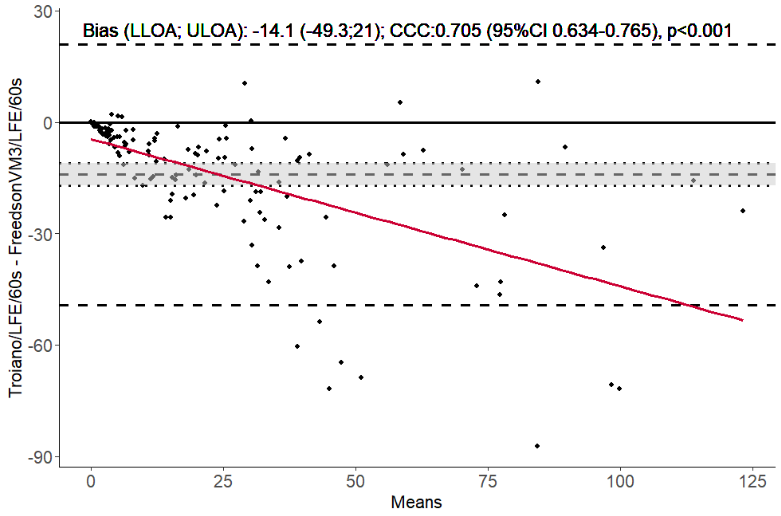

Figure S2: Bland–Altman 95% limits of agreement plot for Troiano and FreedsonVM3 cutoffs, with 60-second epoch and low-frequency extension filter fixed in people with chronic obstructive pulmonary disease (*n*=136). Dashed lines represent bias (mean difference), lower and upper limits of agreement and the grey shadow represent the 95% confidence interval of the mean difference. Bias, lower and upper limits of agreement, Lin's correlation coefficient and the *p*-value for the slope of the linear regression of the differences on averages (red line) are presented. CCC - Lin's concordance correlation coefficient. CI – Confidence interval; LLOA – lower limit of agreement; LFE – low frequency extension filter; ULOA – upper limit of agreement.

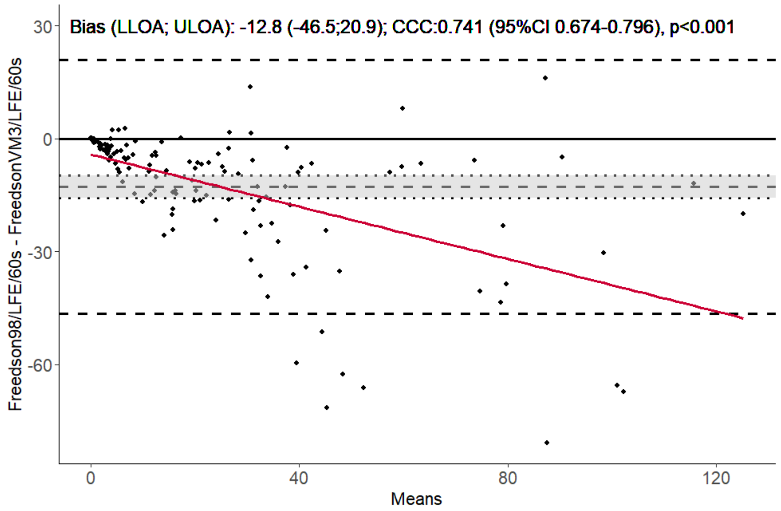

Figure S3: Bland–Altman 95% limits of agreement plot for Freedson98 and FreedsonVM3 cutoffs, with 60-second epoch and low-frequency extension filter fixed in people with chronic obstructive pulmonary disease (*n*=136). Dashed lines represent bias (mean difference), lower and upper limits of agreement and the grey shadow represent the 95% confidence interval of the mean difference. Bias, lower and upper limits of agreement, Lin's correlation coefficient and the *p*-value for the slope of the linear regression of the differences on averages (red line) are presented. CCC - Lin's concordance correlation coefficient. CI – Confidence interval; LLOA – lower limit of agreement; LFE – low frequency extension filter; ULOA – upper limit of agreement.

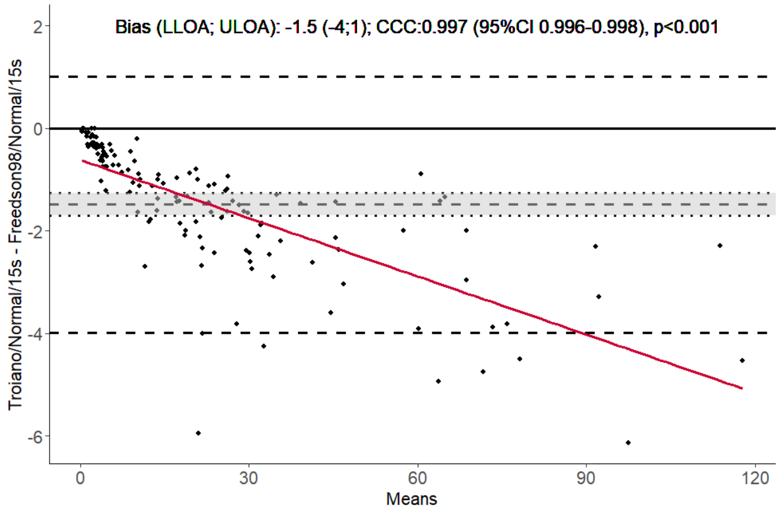

Figure S4: Bland–Altman 95% limits of agreement plot for Troiano and Freedson98 cutoffs, with 15-second epoch and normal filter fixed in people with chronic obstructive pulmonary disease (*n*=136). Dashed lines represent bias (mean difference), lower and upper limits of agreement and the grey shadow represent the 95% confidence interval of the mean difference. Bias, lower and upper limits of agreement, Lin's correlation coefficient and the *p*-value for the slope of the linear regression of the differences on averages (red line) are presented. CCC - Lin's concordance correlation coefficient. CI – Confidence interval; LLOA – lower limit of agreement; ULOA – upper limit of agreement.

.

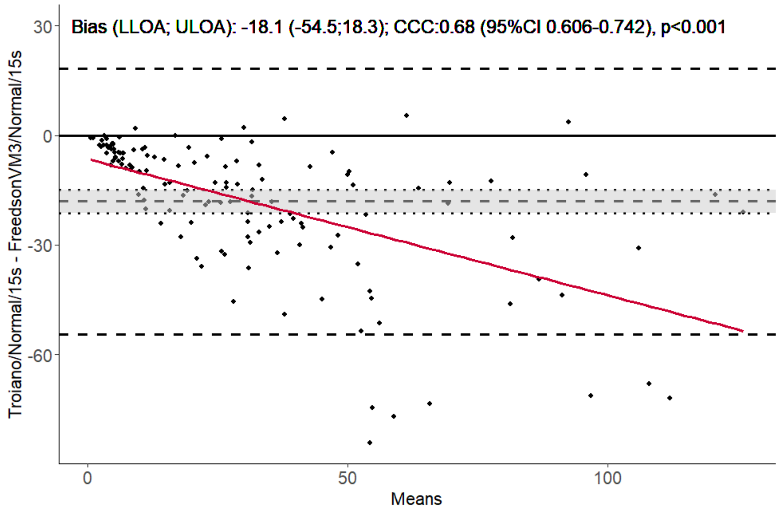

Figure S5: Bland–Altman 95% limits of agreement plot for Troiano and FreedsonVM3 cutoffs, with 15-second epoch and normal filter fixed in people with chronic obstructive pulmonary disease (*n*=136). Dashed lines represent bias (mean difference), lower and upper limits of agreement and the grey shadow represent the 95% confidence interval of the mean difference. Bias, lower and upper limits of agreement, Lin's correlation coefficient and the *p*-value for the slope of the linear regression of the differences on averages (red line) are presented. CCC - Lin's concordance correlation coefficient. CI – Confidence interval; LLOA – lower limit of agreement; ULOA – upper limit of agreement.

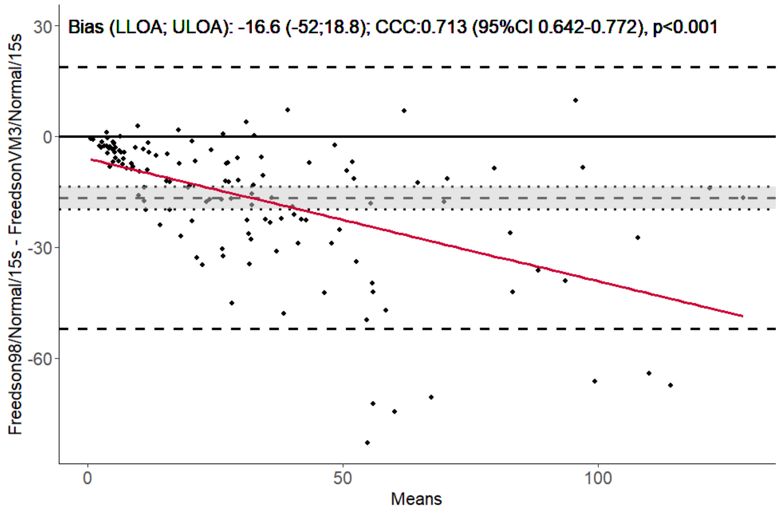

Figure S6: Bland–Altman 95% limits of agreement plot for Freedson98 and FreedsonVM3 cutoffs, with 15-second epoch and normal filter fixed in people with chronic obstructive pulmonary disease (*n*=136). Dashed lines represent bias (mean difference), lower and upper limits of agreement and the grey shadow represent the 95% confidence interval of the mean difference. Bias, lower and upper limits of agreement, Lin's correlation coefficient and the *p*-value for the slope of the linear regression of the differences on averages (red line) are presented. CCC - Lin's concordance correlation coefficient. CI – Confidence interval; LLOA – lower limit of agreement; ULOA – upper limit of agreement.

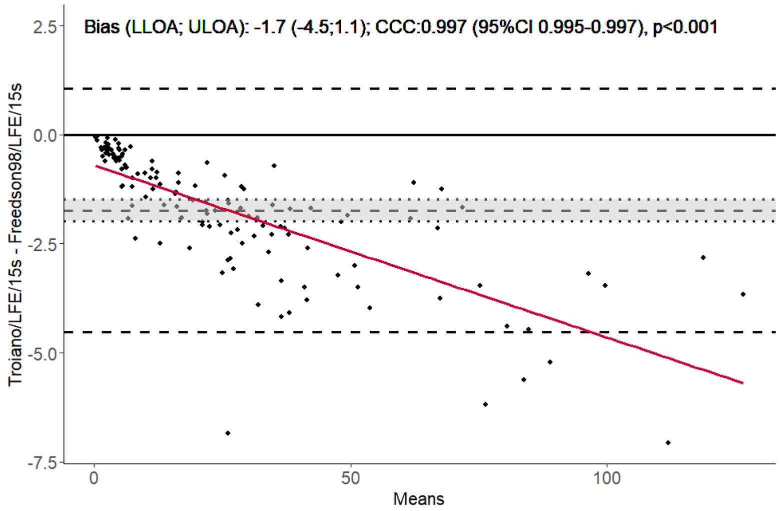

Figure S7: Bland–Altman 95% limits of agreement plot for Troiano and Freedson98 cutoffs, with 15-second epoch and low-frequency extension filter fixed in people with chronic obstructive pulmonary disease (*n*=136). Dashed lines represent bias (mean difference), lower and upper limits of agreement and the grey shadow represent the 95% confidence interval of the mean difference. Bias, lower and upper limits of agreement, Lin's correlation coefficient and the *p*-value for the slope of the linear regression of the differences on averages (red line) are presented. CCC - Lin's concordance correlation coefficient. CI – Confidence interval; LLOA – lower limit of agreement; LFE – low frequency extension filter; ULOA – upper limit of agreement.

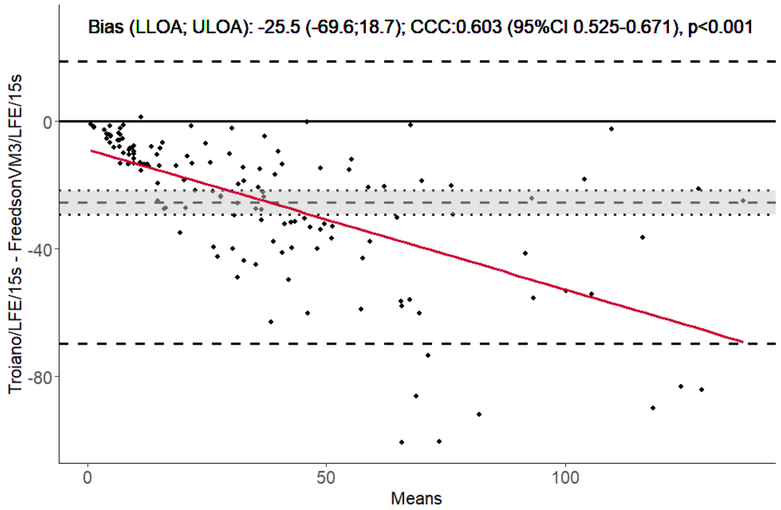

Figure S8: Bland–Altman 95% limits of agreement plot for Troiano and FreedsonVM3 cutoffs, with 15-second epoch and low-frequency extension filter fixed in people with chronic obstructive pulmonary disease (*n*=136). Dashed lines represent bias (mean difference), lower and upper limits of agreement and the grey shadow represent the 95% confidence interval of the mean difference. Bias, lower and upper limits of agreement, Lin's correlation coefficient and the *p*-value for the slope of the linear regression of the differences on averages (red line) are presented. CCC - Lin's concordance correlation coefficient. CI – Confidence interval; LLOA – lower limit of agreement; LFE – low frequency extension filter; ULOA – upper limit of agreement.

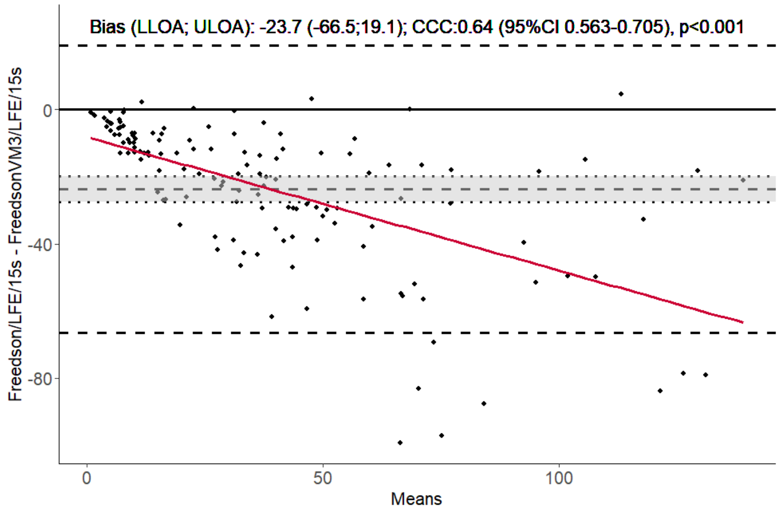

Figure S9: Bland–Altman 95% limits of agreement plot for Freedson98 and FreedsonVM3 cutoffs, with 15-second epoch and low-frequency extension filter fixed in people with chronic obstructive pulmonary disease (*n*=136). Dashed lines represent bias (mean difference), lower and upper limits of agreement and the grey shadow represent the 95% confidence interval of the mean difference. Bias, lower and upper limits of agreement, Lin's correlation coefficient and the *p*-value for the slope of the linear regression of the differences on averages (red line) are presented. CCC - Lin's concordance correlation coefficient. CI – Confidence interval; LLOA – lower limit of agreement; LFE – low frequency extension filter; ULOA – upper limit of agreement.

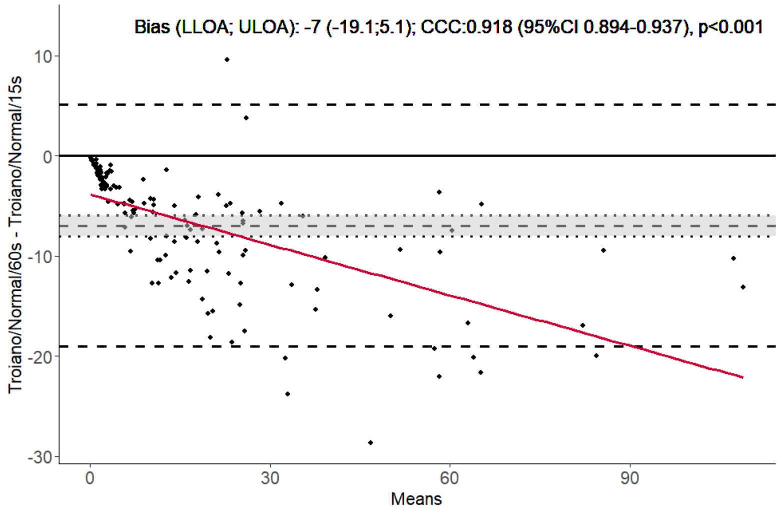

Figure S10: Bland–Altman 95% limits of agreement plot for 60-second and 15-second epochs, with Troiano cutoff and normal filter fixed in people with chronic obstructive pulmonary disease (*n*=136). Dashed lines represent bias (mean difference), lower and upper limits of agreement and the grey shadow represent the 95% confidence interval of the mean difference. Bias, lower and upper limits of agreement, Lin's correlation coefficient and the *p*-value for the slope of the linear regression of the differences on averages (red line) are presented. CCC - Lin's concordance correlation coefficient. CI – Confidence interval; LLOA – lower limit of agreement; ULOA – upper limit of agreement.

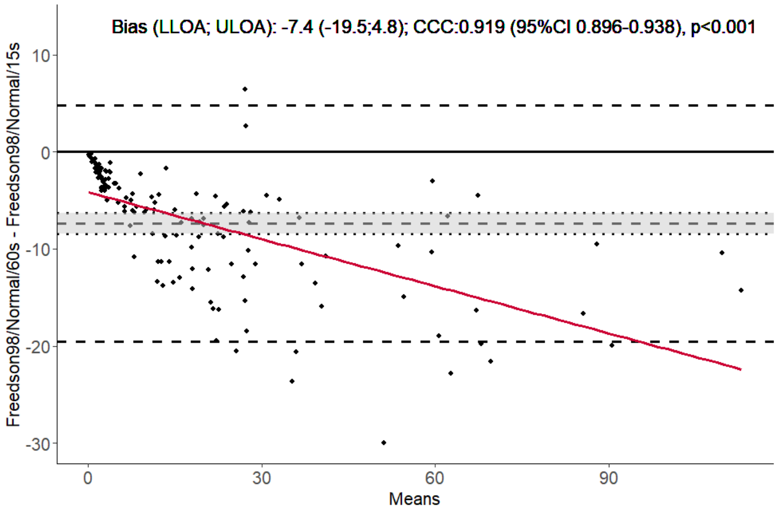

Figure S11: Bland–Altman 95% limits of agreement plot for <u>60-second and 15-second epochs</u>, with Freedson98 cutoff and normal filter fixed in people with chronic obstructive pulmonary disease (*n*=136). Dashed lines represent bias (mean difference), lower and upper limits of agreement and the grey shadow represent the 95% confidence interval of the mean difference. Bias, lower and upper limits of agreement, Lin's correlation coefficient and the *p*-value for the slope of the linear regression of the differences on averages (red line) are presented. CCC - Lin's concordance correlation coefficient. CI – Confidence interval; LLOA – lower limit of agreement; ULOA – upper limit of agreement.

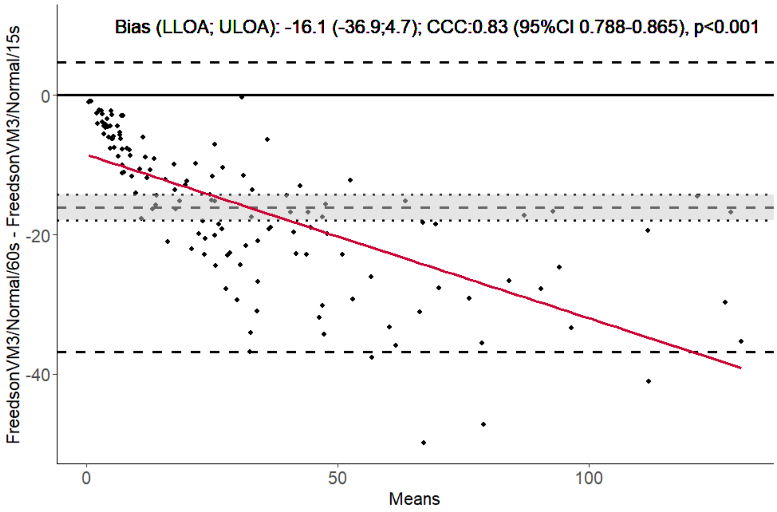

Figure S12: Bland–Altman 95% limits of agreement plot for <u>60-second and 15-second epochs</u>, with FreedsonVM3 cutoff and normal filter fixed in people with chronic obstructive pulmonary disease (*n*=136). Dashed lines represent bias (mean difference), lower and upper limits of agreement and the grey shadow represent the 95% confidence interval of the mean difference. Bias, lower and upper limits of agreement, Lin's correlation coefficient and the *p*-value for the slope of the linear regression of the differences on averages (red line) are presented. CCC - Lin's concordance correlation coefficient. CI – Confidence interval; LLOA – lower limit of agreement; ULOA – upper limit of agreement.

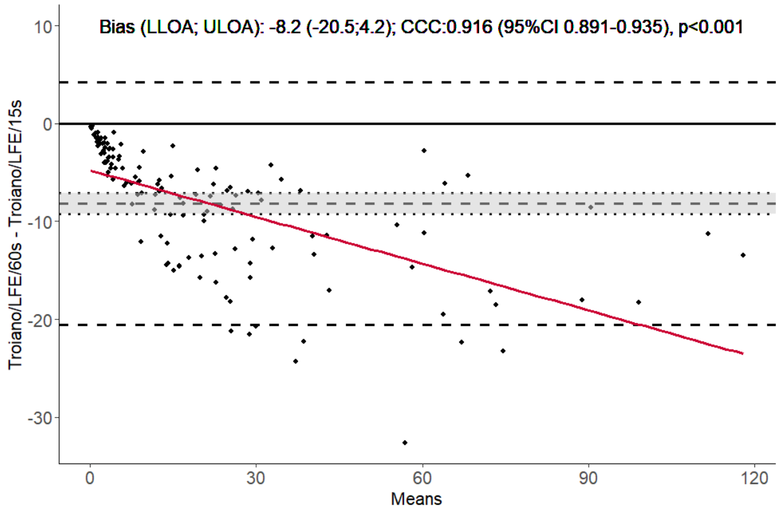

Figure S13: Bland–Altman 95% limits of agreement plot for 60-second and 15-second epochs, with Troiano cutoff and low frequency extension filter fixed in people with chronic obstructive pulmonary disease (*n*=136). Dashed lines represent bias (mean difference), lower and upper limits of agreement and the grey shadow represent the 95% confidence interval of the mean difference. Bias, lower and upper limits of agreement, Lin's correlation coefficient and the *p*-value for the slope of the linear regression of the differences on averages (red line) are presented. CCC - Lin's concordance correlation coefficient. CI – Confidence interval; LLOA – lower limit of agreement; LFE – low frequency extension filter; ULOA – upper limit of agreement.

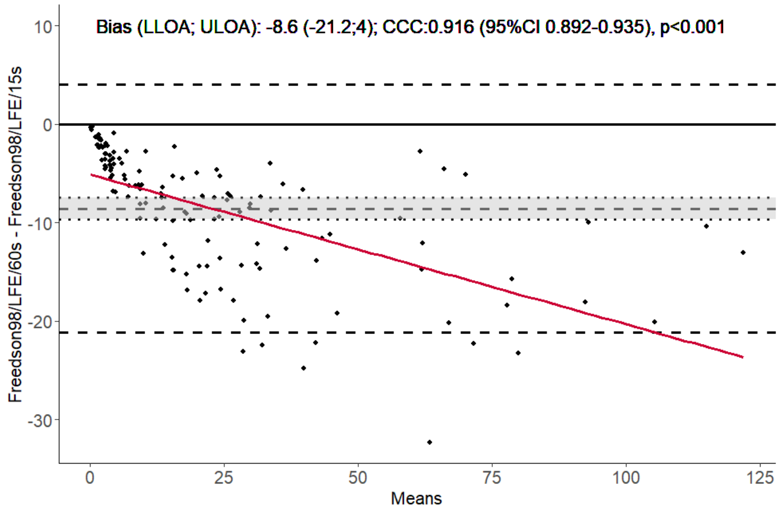

Figure S14: Bland–Altman 95% limits of agreement plot for 60-second and 15-second epochs, with Freedson98 cutoff and low frequency extension filter fixed in people with chronic obstructive pulmonary disease (*n*=136). Dashed lines represent bias (mean difference), lower and upper limits of agreement and the grey shadow represent the 95% confidence interval of the mean difference. Bias, lower and upper limits of agreement, Lin's correlation coefficient and the *p*-value for the slope of the linear regression of the differences on averages (red line) are presented. CCC - Lin's concordance correlation coefficient. CI – Confidence interval; LLOA – lower limit of agreement; LFE – low frequency extension filter; ULOA – upper limit of agreement.

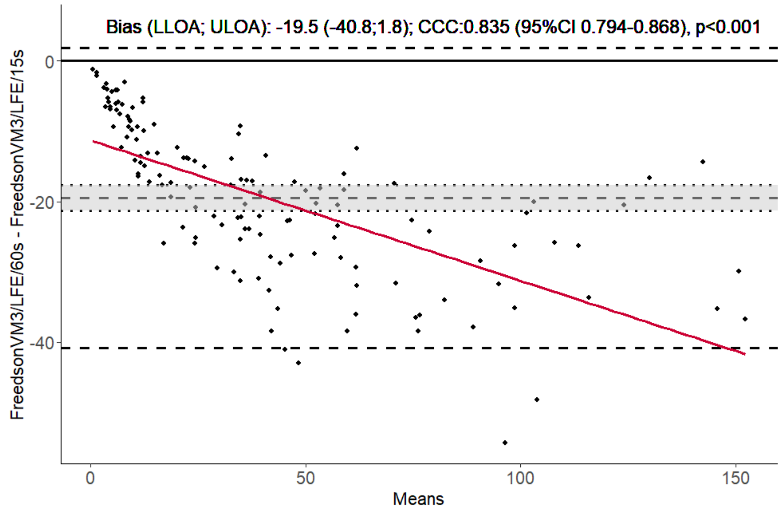

Figure S15: Bland–Altman 95% limits of agreement plot for 60-second and 15-second epochs, with FreedsonVM3 cutoff and low frequency extension filter fixed in people with chronic obstructive pulmonary disease (*n*=136). Dashed lines represent bias (mean difference), lower and upper limits of agreement and the grey shadow represent the 95% confidence interval of the mean difference. Bias, lower and upper limits of agreement, Lin's correlation coefficient and the *p*-value for the slope of the linear regression of the differences on averages (red line) are presented. CCC - Lin's concordance correlation coefficient. CI – Confidence interval; LLOA – lower limit of agreement; LFE – low frequency extension filter; ULOA – upper limit of agreement.

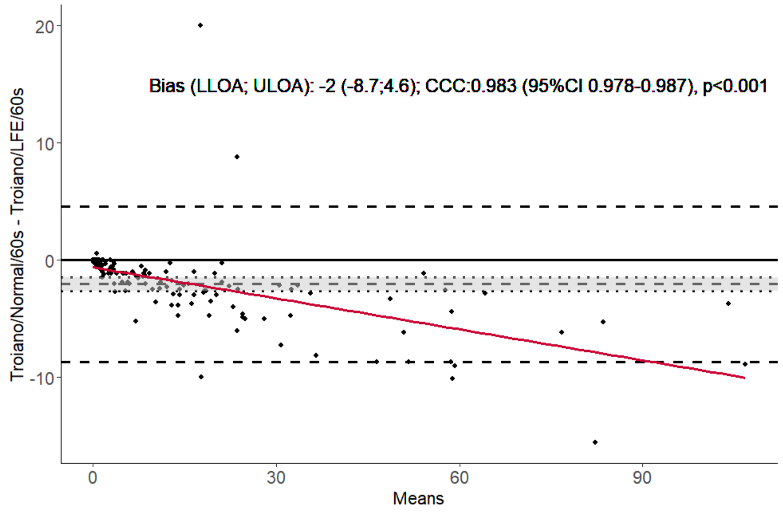

Figure S16: Bland–Altman 95% limits of agreement plot for normal and low-frequency extension filters, with Troiano cutoff and 60-second epochs fixed in people with chronic obstructive pulmonary disease (*n*=136). Dashed lines represent bias (mean difference), lower and upper limits of agreement and the grey shadow represent the 95% confidence interval of the mean difference. Bias, lower and upper limits of agreement, Lin's correlation coefficient and the *p*-value for the slope of the linear regression of the differences on averages (red line) are presented. CCC - Lin's concordance correlation coefficient. CI – Confidence interval; LLOA – lower limit of agreement; LFE – low frequency extension filter; ULOA – upper limit of agreement.

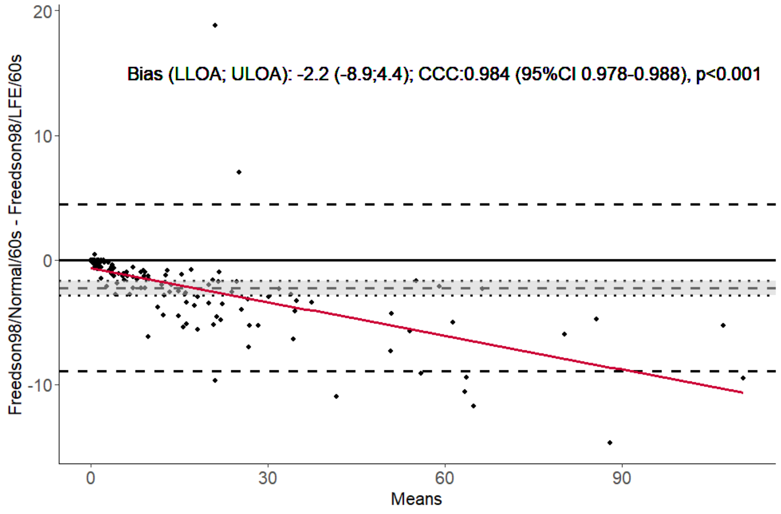

Figure S17: Bland–Altman 95% limits of agreement plot for <u>normal and low-frequency extension filters</u>, with Freedson98 cutoff and 60-second epochs fixed in people with chronic obstructive pulmonary disease (*n*=136). Dashed lines represent bias (mean difference), lower and upper limits of agreement and the grey shadow represent the 95% confidence interval of the mean difference. Bias, lower and upper limits of agreement, Lin's correlation coefficient and the *p*-value for the slope of the linear regression of the differences on averages (red line) are presented. CCC - Lin's concordance correlation coefficient. CI – Confidence interval; LLOA – lower limit of agreement; LFE – low frequency extension filter; ULOA – upper limit of agreement.

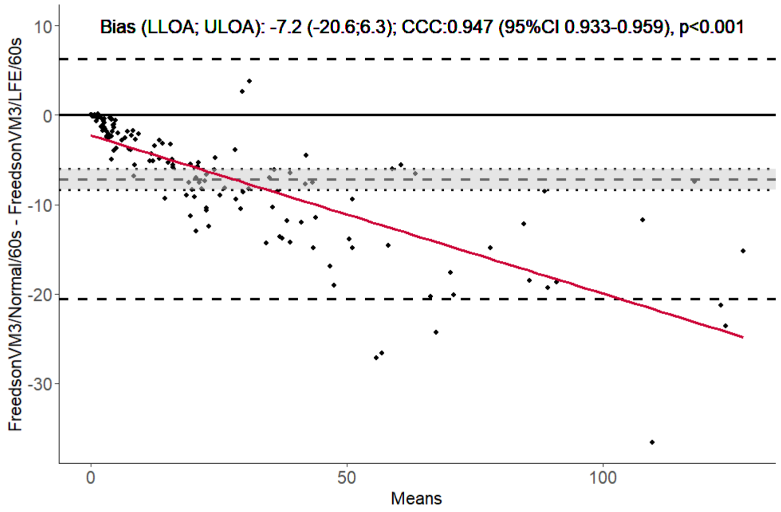

Figure S18: Bland–Altman 95% limits of agreement plot for <u>normal and low-frequency extension filters</u>, with FreedsonVM3 cutoff and 60-second epochs fixed in people with chronic obstructive pulmonary disease (*n*=136). Dashed lines represent bias (mean difference), lower and upper limits of agreement and the grey shadow represent the 95% confidence interval of the mean difference. Bias, lower and upper limits of agreement, Lin's correlation coefficient and the *p*-value for the slope of the linear regression of the differences on averages (red line) are presented. CCC - Lin's concordance correlation coefficient. CI – Confidence interval; LLOA – lower limit of agreement; LFE – low frequency extension filter; ULOA – upper limit of agreement.

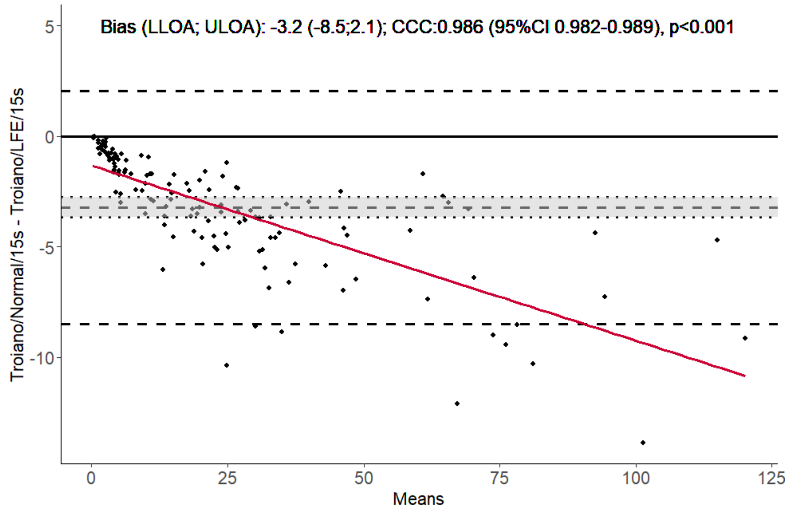

Figure S19: Bland–Altman 95% limits of agreement plot for normal and low-frequency extension filters, with Troiano cutoff and 15-second epochs fixed in people with chronic obstructive pulmonary disease (*n*=136). Dashed lines represent bias (mean difference), lower and upper limits of agreement and the grey shadow represent the 95% confidence interval of the mean difference. Bias, lower and upper limits of agreement, Lin's correlation coefficient and the *p*-value for the slope of the linear regression of the differences on averages (red line) are presented. CCC - Lin's concordance correlation coefficient. CI – Confidence interval; LLOA – lower limit of agreement; LFE – low frequency extension filter; ULOA – upper limit of agreement.

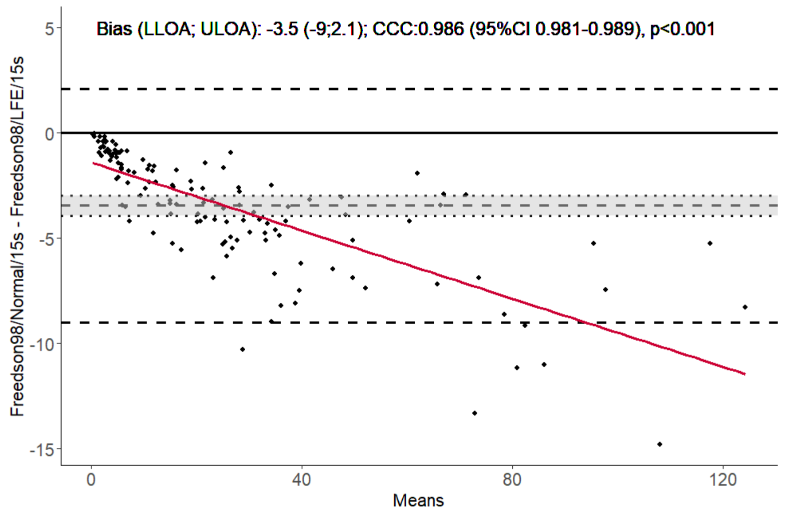

Figure S20: Bland–Altman 95% limits of agreement plot for normal and low-frequency extension filters, with Freedson98 cutoff and 15-second epochs fixed in people with chronic obstructive pulmonary disease (*n*=136). Dashed lines represent bias (mean difference), lower and upper limits of agreement and the grey shadow represent the 95% confidence interval of the mean difference. Bias, lower and upper limits of agreement, Lin's correlation coefficient and the *p*-value for the slope of the linear regression of the differences on averages (red line) are presented. CCC - Lin's concordance correlation coefficient. CI – Confidence interval; LLOA – lower limit of agreement; LFE – low frequency extension filter; ULOA – upper limit of agreement.

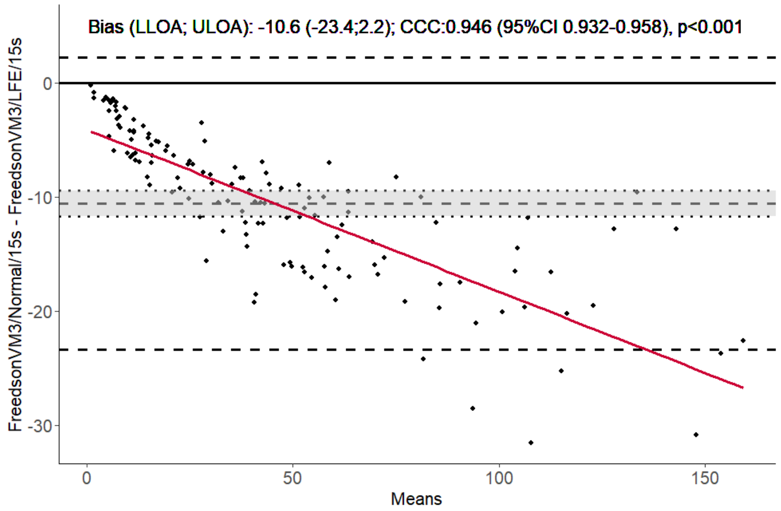

Figure S21: Bland–Altman 95% limits of agreement plot for <u>normal and low-frequency extension filters</u>, with FreedsonVM3 cutoff and 15-second epochs fixed in people with chronic obstructive pulmonary disease (*n*=136). Dashed lines represent bias (mean difference), lower and upper limits of agreement and the grey shadow represent the 95% confidence interval of the mean difference. Bias, lower and upper limits of agreement, Lin's correlation coefficient and the *p*-value for the slope of the linear regression of the differences on averages (red line) are presented. CCC - Lin's concordance correlation coefficient. CI – Confidence interval; LLOA – lower limit of agreement; LFE – low frequency extension filter; ULOA – upper limit of agreement.
